# Toxicological Mechanisms of Uranium-Induced Apoptosis in HK-2 Cells: A Proteomics and Metabolomics Study

**DOI:** 10.3390/toxics13080699

**Published:** 2025-08-20

**Authors:** Zihuan Wang, Yongxiang Huang, Yue Zhang, Xuejuan Wu, Yuanyuan Yang, Jiayu Song, Kunling Guo, Mingyuan Wang, Junjie Chen, Shirong Qiang

**Affiliations:** 1 Key Laboratory of Preclinical Study for New Drugs of Gansu Province, Institute of Physiology, School of Basic Medical Sciences, Lanzhou University, Lanzhou 730000, China; 220220926500@lzu.edu.cn (Z.W.); zhangyue23@lzu.edu.cn (Y.Z.); wuxj2024@lzu.edu.cn (X.W.); yangyy2024@lzu.edu.cn (Y.Y.); 2 The First School of Clinical Medicine, Lanzhou University, Lanzhou 730000, China; yxhuang2021@lzu.edu.cn (Y.H.); wmingyuan2023@lzu.edu.cn (M.W.); 3 Northwest Institute of Eco-Environment and Resources, Chinese Academy of Sciences, Lanzhou 730000, China; songjiayu21@mail.ucas.ac.cn (J.S.); chenjunjie241@mails.ucas.ac.cn (J.C.); 4 School of Public Health, Shanghai Jiao Tong University, Shanghai 200025, China; gkl-200001@sjtu.edu.cn

**Keywords:** uranium, HK-2 cell, apoptosis, proteomics, metabolomics

## Abstract

The rapid development of the nuclear industry and mining has increased environmental radioactive contamination, posing potentially ecological risks and health threats to humans. Uranium compounds are known to exhibit selective nephrotoxicity, but their toxicological processes and mechanisms still remain poorly understood and controversial. In this study, the uranyl-induced toxicity in human renal tubular epithelial cells (HK-2) were explored using flow cytometry, DAPI staining, and comet assays. Our results demonstrate that uranium exposure primarily triggers apoptosis. Kyoto Encyclopedia of Genes and Genomes pathway enrichment and protein–protein interaction (PPI) analyses revealed significant associations with DNA damage. Moreover, aberrant expression of ABC transporters (e.g., ABCB7) and mitochondrial-related proteins confirms uranium-induced mitochondrial dysfunction. Gene Ontology functional annotation implicated extrinsic apoptotic signaling pathways in uranium-induced cell death. The downregulation of the UBL5 protein also pointed to endoplasmic reticulum stress-mediated apoptosis. In summary, uranium exposure can induce the apoptosis of HK-2 cells through intrinsic pathways by damaging DNA and mitochondria and disrupting protein synthesis, with secondary contributions from endoplasmic reticulum stress and extrinsic apoptotic signaling.

## 1. Introduction

Uranium is a naturally ubiquitous actinide radioactive element that is widely distributed in earth’s crust, rocks and soil [1]. It exists in nature in three isotopes: uranium 234 (0.006%), uranium 235 (0.72%), and uranium 238 (99.27%) [2,3]. The development of the nuclear industry and mining industry has greatly increased the likelihood of human exposure to uranium [4,5]. Natural uranium can enter the human body through inhalation, ingestion, or skin contact [2,6,7]. The higher the solubility of uranium compounds, the greater its toxicity generally. The hexavalent uranium is more toxic than the less soluble tetravalent uranium minerals [8,9]. The kidney is the main organ affected by the toxicity of uranium; however, the molecular mechanism of uranium-induced kidney injury has not been fully studied, and there is still a lack of targeted treatment for acute uranium poisoning. Therefore, it is important to further investigate the mechanism of uranium nephrotoxicity. Previous studies have shown that uranium exposure has been shown to inhibit cell viability, induce apoptosis, and enhance the production of reactive oxygen species (ROS) [10]. Since the proximal tubule was identified as the most severely affected site in the organ [11], researchers cultured and examined damage in rat (NRK-52E) [12] and human (HK-2) renal proximal tubular epithelial cells [13,14] in uranium-containing media, indicating that uranium induced caspase-dependent intrinsic apoptosis [15].

Intrinsic apoptosis, also known as the mitochondrial pathway of apoptosis, is where cells sense intracellular stressors (i.e., growth factor or nutrient deprivation, DNA damage) and then kill themselves through a process involving extramitochondrial membrane permeabilization (MOMP) [16,17]. In extrinsic apoptosis, extracellular signals are recognized by death receptors on the plasma membrane and induce death. Death ligands, such as Fas ligands (FASL), tumor necrosis factor (TNF), or TNF-associated apoptosis-inducing ligands (TRAIL), can be recognized by their respective specific cell-surface death receptors, Fas receptor (FasR), TNF receptors 1 and 2 (TNFR1/2), leading to apoptosis [18,19,20]. Yuhui Hao [15] demonstrated that depleted uranium induced the overexpression of soluble FasR (sFasR) and soluble Fas ligand (sFasL) in HK-2 cells and activated extrinsic apoptosis.

As is well known, endoplasmic reticulum stress (ERS) can lead to cell apoptosis. ERS refers to a stress response that occurs within the cell endoplasmic reticulum (ER) when it encounters various stresses and injuries. The process by which ERS triggers cell apoptosis involves the activation of the unfolded protein response (UPR), which initially attempts to restore ER function [21]. However, if the stress persists and is not resolved, it shifts to a pro-apoptotic signal. This transition is achieved through various mechanisms, including the activation of the CHOP protein, which could lead to the suppression of anti-apoptotic proteins and the activation of pro-apoptotic proteins [22]. The activation of death receptor 5 (DR5) initiates the extrinsic apoptotic pathway [23], and the activation of the BH3-interacting-domain death agonist (BID) protein links the extrinsic apoptotic pathway with the intrinsic apoptotic pathway [24]. Finally, it leads to the release of cytochrome c from the mitochondria, the activation of the caspase cascade, and the induction of programmed cell death. Hu et al. [25] found that uranium-induced apoptosis in NRK-52E cells could be attributed to ROS production, ER stress, and downregulation of PI3K/AKT/mTOR signaling; however, the specific mechanism of the apoptosis remains unknown under the uranium exposure.

In this study, for the first time, combined proteomic and metabolomic analysis was used to characterize the nephrotoxicity of uranium and to explore the potential toxic mechanism related to apoptosis. Multi-omics integration analysis techniques can present the three-dimensional biological processes of a system from different molecular levels. Proteomics–metabolomics combined analysis integrates information from the protein and metabolite levels to delve deeper into candidate key factors and provide more evidence for biological mechanisms. Combined with multivariate statistical analysis and joint analysis, the possible metabolic pathways of uranium-induced apoptosis were screened. This study provides a new basis for studying the toxicological mechanism of uranium-induced apoptosis.

## 2. Materials and Methods

### 2.1. Cells Culture and Reagents Preparation

Human renal proximal tubule (HK-2) (CL-0109) were provided by Wuhan Pricella Biotechnology Co., Ltd. HK-2 cells were cultured in a Dulbecco’s Modified Eagle medium (VivaCell, Shanghai, China) supplemented with 10% (*v*/*v*) fetal bovine serum (FBS) (VivaCell, Shanghai, China) and 1% antibiotic (100 U/mL penicillin and 10 μg/mL streptomycin) (VivaCell, Shanghai, China). Cells were seeded in a 10 cm culture dish and incubated in a humidified atmosphere with 5% CO_2_ at 37 °C. The culture medium was renewed every 2 days.

A stock solution of uranyl nitrate (pH 3, 1 M) was prepared with sterile double steamed deionized water. The resulting solution was passed through a 0.22 μm syringe filter and diluted in cell culture media to desired concentrations. The pH of the diluted solution is within the normal physiological range.

### 2.2. Cytotoxicity Assessment of HK-2 Cells

Cell viability was evaluated with Cell Counting Kit-8 (K1018, APExBIO, Houston, TX, USA) strictly following the manufacturer instructions. 7 × 10^3^ HK-2 cells suspended in 100 μL DMEM supplemented with 10% FBS were seeded to each well of a 96-well plate. The cells were incubated in a humidified atmosphere with 5% CO_2_ at 37 °C for 24 h. Then, the culture medium was replaced with fresh DMEM and different concentrations of uranyl ion. After different periods of exposure, the medium was replaced with PBS and the cells were washed. A 100 μL 1:9 mixture of CCK-8 reagent and DMEM was added to each well. After the addition of the CCK-8 reagent, the plate was incubated at 37 °C for 1 h. The absorbance at 450 nm was measured using a fully functional Synergy NEO2 (Agilent, Santa Clara, CA, USA) microplate reader. Cell viability was calculated as the ratio between absorbances of treated wells and untreated control wells (All data were subtracted from the blank group mean). While the micromolar concentrations applied here exceed typical environmental exposures, they are within the range that can be reached in kidneys following high-end chronic intake or acute poisoning events, and are therefore justified for hazard identification and mode-of-action studies.

### 2.3. Flow Cytometry

HK-2 cells were seeded in a 6 cm culture dish for 24 h and then incubated with different concentrations of uranyl nitrate for 24 h. The medium was aspirated and placed in a centrifuge tube. The cells were carefully digested after washing with PBS. The medium, PBS, and cell suspension were mixed and centrifuged at 1000 RPM for 5 min. Cells were subsequently washed with PBS, and 5 × 10^4^ cells were taken for subsequent staining. Flow cytometry was performed after ice bath staining for 15 to 20 min.

HK-2 cells exposed to different concentrations of uranyl nitrate were stained with Annexin Ⅴ/PI dye (Uelandy, Suzhou, China). Where the Q1 quadrant is mechanical damage/cell debris, Q2 is late apoptotic and necrotic cells, Q3 is early apoptotic cells, and Q4 is normal living cells [13].

HK-2 cells exposed to different concentrations of uranyl nitrate were stained with PI dye, and cell apoptosis was judged according to the subG1 peak before G1 cycle.

### 2.4. AO/PI Staining

HK-2 cells were seeded in 6-well plates and treated with uranium for 24 h before AO/PI staining (Bestbio, Shanghai, China). After washing with PBS, cells were stained with acridine orange (AO) and propidium iodide (PI) mixture for 20 min. The color of cells was observed at 488 nm under a fluorescence microscope. The normal cells showed green and yellow-green with normal morphology and structure. The apoptotic cells had condensed chromatin and fragmented nuclei with yellow or orange color; however, the necrotic cells exhibit a red color.

### 2.5. Comet Assay

The cells were treated with different concentrations of uranyl ion and incubated for 24 h or 48 h. Glass slides were preheated at 50 °C, and 100 μL of 1% agarose was applied to a slide, followed by immediate cooling at 4 °C for 10 min. The 10 μL cells (1 × 10^4^) were mixed with 75 μL of low-melting agarose (0.7%) and were layered over the base agarose. After solidification, another layer of low-melting agarose (75 μL, 0.7%) was added and the slides were cooled for 30 min. Subsequently, the slides were immersed in ice-cold lysis solution for 2 h, and were immersed in electrophoresis buffer (1 mmol/L EDTA, 300 mmol/L NaOH) for 40 min. Then electrophoresis was performed at 25 V for 20 min. After the completion of electrophoresis, slides were washed with PBS buffer for 10 min × 3 at 4 °C, then 20 μL of PI was added and a coverslip was placed over it and observed under a fluorescent microscope. Then, fluorescent microscopy images were taken (KFS210, Beijing BioRab Technology Co., Ltd., Beijing, China).

### 2.6. DAPI Staining

HK-2 cells were seeded in 6-well plates, exposed to uranyl nitrate for 24 h, washed with PBS, and then stained with DAPI for 10 min. The morphology of stained cells was observed under a fluorescence-inverted microscope after washing with PBS.

### 2.7. Metabolomic Sequencing and Analysis

Samples were transferred to EP tubes in triplicate (300 μL, 300 μL, 400 μL) with 1000 μL of extract containing internal standard (methanol acetonitrile volume ratio = 1:1, internal standard concentration 20 mg/L) and vortexed and mixed for 30 s. After that, steel balls were added, processed with a 45 Hz grinder for 10 min, and sonicated for 10 min (ice water bath). After standing at −20 °C for one hour, the samples were centrifuged at 12,000 rpm for 15 min at 4 °C. After that, 500 μL of supernatant was removed in EP tubes, and the extract was dried in a vacuum concentrator. Mass spectrometry analysis was performed by Beijing Biomarker Technology Co., LTD. (Beijing, China). The LC-MS system for metabolomics analysis consisted of a Waters Acquity I-Class PLUS ultra-high-performance liquid chromatography coupled to a Waters Xevo G2-XS QTOF high-resolution mass spectrometer. The column used was Acquity UPLC HSS T3 column (1.8 μm 2.1 × 100 mm) purchased from Waters (Milford, MA, USA). The original data collected by MassLynx V4.2 were processed by Progenesis QI software 2.0 (Waters, Milford, MA, USA) for peak extraction, peak alignment, and other data processing, and were identified based on the online METLIN database of Progenesis QI software 2.0, public database and self-built database of Bmaike.

### 2.8. Proteomics Sequencing and Analysis

A total of 300 μL of 8 M urea was added to the sample, and the protease inhibitor was added at 10% of the lysate. After centrifuging at 14,100× *g* for 20 min, the supernatant was collected. The protein concentration was determined using the Bradford method, and the rest was frozen to −80 °C. A total of 50 µg aliquot of extracted proteins from each sample was then subjected to reduction, by adding 200 mM dithiothreitol (DTT) solution and incubating at 37 °C for 1 h. The sample was diluted 8 times by adding 50 mM ammonium bicarbonate (ABC) buffer. Then, trypsin was added (trypsin/protein = 1:25) and the sample was incubated at 37 °C overnight. Protein solutions were separated using reversed-phase separation. Label-free mass spectrometry was performed on a RIGOL L-3000 HPLC system (Beijing Puyuan Varitronics Technology Co., Ltd., Beijing, China) using a Thermo Orbitrap Fusion mass spectrometer (Thermo Fisher Scientific, San Jose, CA, USA). The database used was Uniprot_HUMAN (downloaded on 2019.4.20). Maxquant 1.5.2.8 software was used to process the obtained MS/MS data.

### 2.9. Statistical Analysis

The data in this article are the average values ± SD of the results obtained from at least three repeated measurements. The analysis was performed using GraphPad Prism 9.5 (GraphPad Software, La Jolla, CA, USA). Differences between groups were analyzed using one-way ANOVA followed by Turkey’s multiple comparisons tests. When *p* < 0.05, it was considered that there was a significant difference between the groups.

The data were pre-processed by normalizing the total peak intensity values and Support Vector Regression (SVR) correction. The normalized data were analyzed by the R package 3.6.1 (ropls), in which multivariate data analysis was performed, including principal component analysis (PCA) and orthogonal partial least squares discriminant analysis (OPLS-DA). Seven-fold cross-validation and response reciprocity tests were used to assess the robustness of the model. The multidimensional statistics were normalized using the Par normalization method. The importance of each variable in the OPLS-DA model in the prediction (VIP) values were calculated to indicate their contribution to the classification. Student’s *t*-test was applied to determine the significance of differences between two independent samples. *p*-values were obtained from the *t*-test. VIP ≥ 1 and *p*-values < 0.05 were used to screen for metabolites with significant changes. The differentially expressed proteins were screened by fold-change FC, and the differentially expressed proteins were defined as FC > 2 and *p* < 0.01. Pearson correlation analysis was performed to determine the correlation between the two variables. Analysis was performed using BMK Cloud (www.biocloud.net).

## 3. Results

### 3.1. Cytotoxicity Evaluation

**Figure 1:**
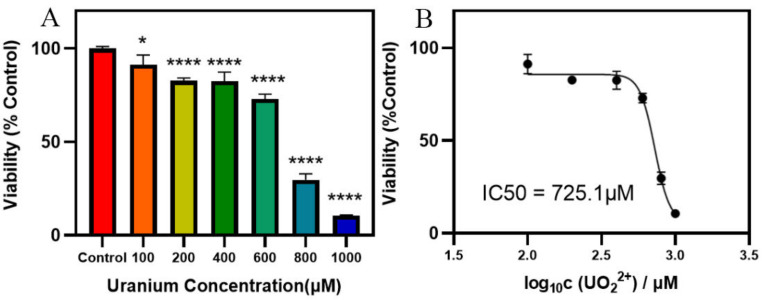
Uranium-reduced cell viability in HK-2 cells. (**A**) Cells were treated with different uranium level for 24 h, and viability was examined with the CCK-8 method. * *p* < 0.05, **** *p* < 0.0001. (**B**) The IC_50_ value of HK-2 cells after uranium treatment was 725.1 μM.

CCK-8 assay was used to detect the toxic effect of uranium on human proximal renal tubular epithelial cells (HK-2) within 24 h. As shown in Figure 1A, cell survival decreased in a strictly concentration-dependent manner: The survival rates of 100 μM, 200 μM, 400 μM, 600 μM, 800 μM, and 1000 μM uranium were 91.27%, 82.70%, 82.46%, 72.91%, 29.62%, and 10.56%, respectively, relative to the solvent control (100% survival rate). The nonlinear regression yielded an IC_50_ value of 725.1 μM (Figure 1B). Taken together, these data suggest that uranium produces a potent, concentration-proportional cytotoxic effect on HK-2 cells within one day of exposure.

### 3.2. Uranyl Nitrate Caused Apoptosis in HK-2 Cells

**Figure 2:**
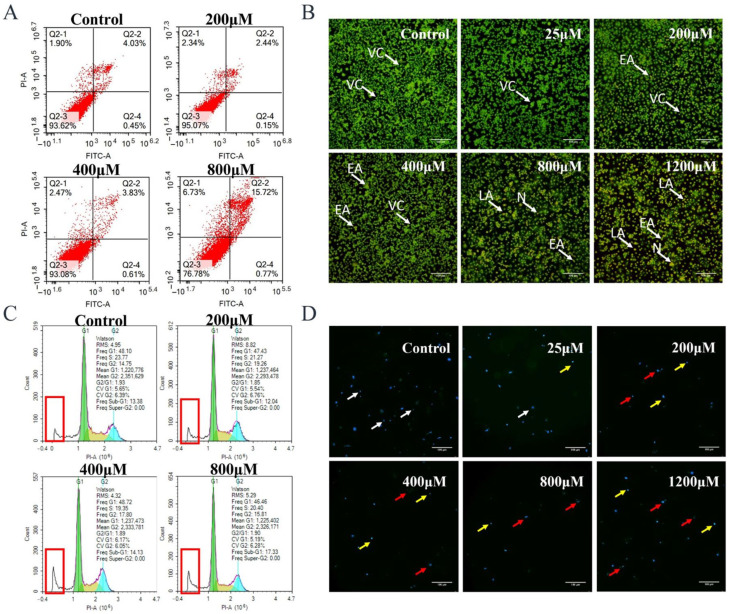
Toxicity of uranyl nitrate on HK-2 cells. (**A**) Flow cytometry with annexin V/PI. (**B**) AO/PI fluorescence staining. Green cells were viable cells (VC), yellow cells were early apoptotic cells (EA), orange cells were late apoptotic cells (LA), and red cells were necrotic cells (N). (**C**) Cell cycle of HK-2 cells after uranyl nitrate exposure. The sub-G1 peak is marked in red boxes. (**D**) DAPI staining. The yellow arrows indicate nucleoplasmic aggregation, the red arrows indicate apoptotic bodies, and the living cells are marked by white arrows. The ruler length was 100 μM.

Figure 2A shows that the annexin V-FITC/PI flow cytometry spot pattern for the Q2-2 quadrant (annexin V^+^/PI^−^, late apoptosis plus secondary necrosis) increased from 4.03% in the control group to 15.72% at 800 µM uranium, respectively. The corresponding Q2-4 quadrant (annexin V^+^/PI^−^, early apoptosis) increased from 0.45% to 0.77% at the same concentration range. This indicates that uranium exposure causes cell death, which may be associated with late apoptosis and necrosis.

To confirm the flow cytometry results, AO/PI dual fluorescence microscopy was performed (Figure 2B). Live cells with intact membranes showed bright green fluorescence, whereas early apoptotic cells with asymmetric membranes showed yellow-orange cytoplasmic staining, whereas cells with fully permeable membranes showed red staining (necrotic or late apoptotic). Figure 2B shows that the proportion of viable cells decreases when uranium is 800 µM. At the same time, orange-yellow cells increased, while red cells accounted for less. This indicates that apoptosis rather than primary necrosis is the predominant mode of death.

The sub-G1 peak in Figure 2C indicates the occurrence of apoptotic cells, more apoptotic cells were observed in the high uranium concentration group than in the low uranium concentration group, indicating that the apoptosis was aggravated at the concentration of 400–800 μM.

In order to further confirm that uranium exposure can lead to cell apoptosis, DAPI staining was performed. As shown in Figure 2D, the yellow arrow indicates nucleoplasmic condensation, the red arrow indicates apoptotic bodies, and the white arrow indicates living cells. The results showed uranium could lead to apoptosis, and the proportion of apoptotic cells increased with the increase of uranium concentration.

### 3.3. Genetic Damage Induced by Uranium in HK-2 Cells

**Figure 3:**
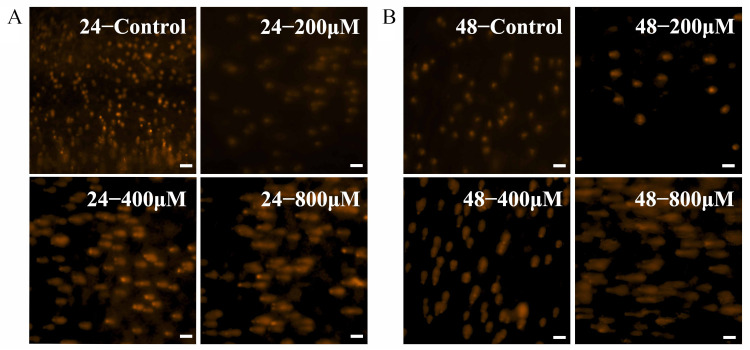
Comet assay. Uranium was exposed for 24 h (**A**) and 48 h (**B**), respectively. The ruler length was 100 μm.

Figure 3 shows that with the increase of uranium level from 0 μM to 800 μM, the degree of DNA damage in cells is aggravated. The comet tail in Figure 3B was longer than that in Figure 3A. The results indicated that the DNA damage of HK-2 cells was more severe with the higher exposure concentration and the longer uranium exposure time.

### 3.4. Differentially Expressed Proteins in Proteomics

**Figure 4:**
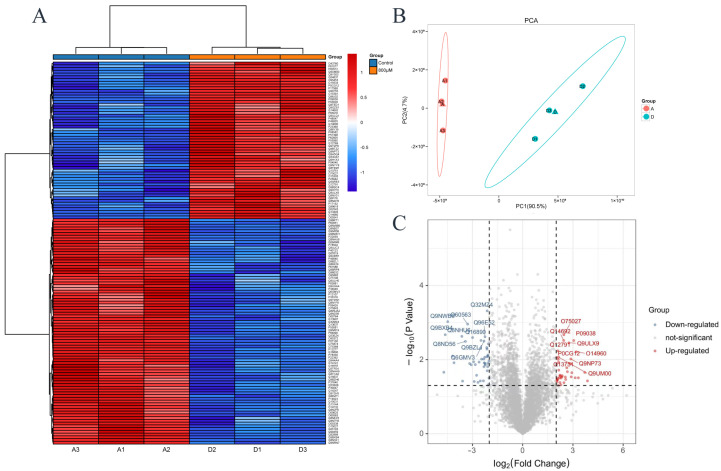
Proteomics of differentially expressed proteins in HK-2 cells exposed to 800 μM uranyl nitrate. (**A**) Clustering heat map. (**B**) PCA. (**C**) Volcano plot.

Figure 4 shows that there are proteomic differences between the control group and the experimental group in which the uranium concentration is 800 μM. In Figure 4A, columns represent different samples, the lines represent different proteins; red means a high expression of protein, blue means a lower expression of protein. The clustering heat map show that there is a large difference between the control group and the experimental group. As can be seen from the volcano plots in Figure 4C, the blue dots are downregulated differential proteins, and the red dots are upregulated differential proteins. Altogether, there were 159 differentially expressed proteins (DEPs), among which 57 were upregulated differentially expressed proteins and 82 were downregulated. The top 15 differentially expressed proteins were summarized as shown in Table 1. According to literature research, most of the top 15 differentially expressed proteins were related to apoptosis [26,27,28,29,30,31,32]. For example, Arglu1 (arginine- and glutamine-rich protein 1) deficiency leads to the upregulation of p53 protein expression and apoptosis in CKO mice [33]. ABCB7 (ATP-binding cassette subfamily B member 7) knockdown inhibits the TGF-β signaling pathway, inhibits the survival of esophageal cancer cells by inducing cell death [34]. Among them, UBL5 (ubiquitin-like protein 5) is the most critical protein related to ERS-induced apoptosis.

### 3.5. KEGG Enrichment Analysis in Proteomics

**Figure 5:**
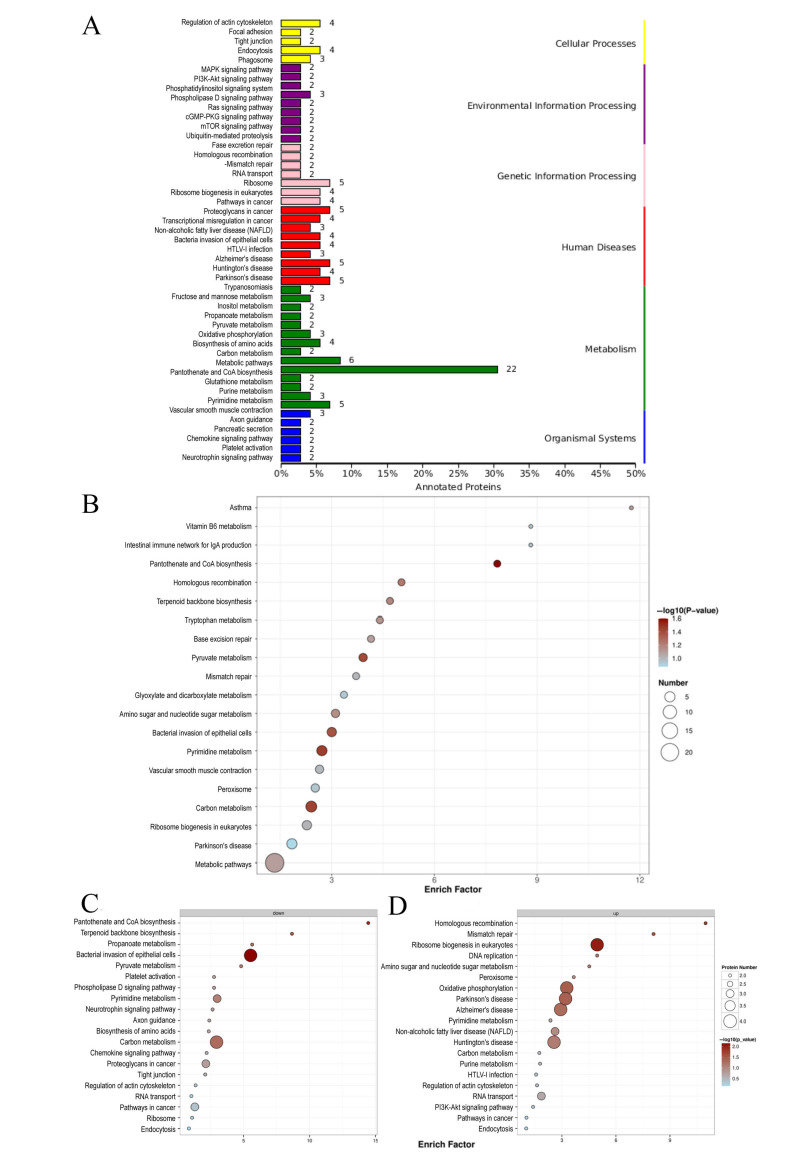
KEGG difference for protein enrichment of annotation and KEGG pathway enrichment map. (**A**) Differential protein KEGG pathway classification bar chart. (**B**) The KEGG pathway enrichment map (summary). (**C**) The KEGG pathway enrichment map (downregulated DEPs). (**D**) The KEGG pathway enrichment map (upregulated DEPs).

Kyoto Encyclopedia of Genes and Genomes (KEEG) enrichment analysis was performed, which could analyze the differentially expressed proteins in different pathways. Figure 5A is the enrichment map of differential protein annotation, showing the pathway overview map of the distribution of all differential proteins. As shown on the right, differentially expressed proteins were mainly concentrated in metabolism, genetic information processing, and human diseases. Specifically, there were more differential proteins related to the total metabolism pathway, carbon metabolism pathway, pyrimidine purine metabolism pathway, cancer-related pathway, and the RNA transport, ribosome, and cellular endocytosis pathways. Purine and pyrimidine metabolism is the core component of nucleic acid molecules and plays a key role in biological processes such as maintaining DNA and RNA structure, gene expression, and energy transfer. It indicates that uranium exposure will cause metabolic disorders in HK-2 cells, leading to abnormal substance synthesis, such as abnormal protein synthesis. Uranium exposure may also have an effect on energy transfer. Enrichment of the endocytosis pathway in cells indicates that uranium may enter HK-2 cells through endocytosis and then cause further damage [35].

Figure 5B is the KEGG pathway enrichment map, and the top 20 differentially expressed proteins with enrichment factors were selected for mapping. The most significant pathways, sorted by *p*-value in Figure 5B, are carbon metabolism, pantothenic acid and coenzyme A synthesis, pyruvate metabolism, and pyrimidine metabolism, respectively. The downregulated differentially expressed proteins were mainly in the pantothenic acid and coenzyme A biosynthesis pathway, terpenoid backbone biosynthesis pathway, pyruvate metabolism pathway, bacterial invasion of epithelial cells pathway, and propionate metabolism pathway [36]. The upregulated differentially expressed proteins are mainly concentrated in pathways such as homologous recombination, mismatch repair, ribosome biogenesis in eukaryotes, DNA replication, and oxidative phosphorylation. Pantothenic acid is a type of vitamin B and a precursor of coenzyme A that is involved in energy metabolism and substance synthesis. Pyruvate is an important intermediate product in metabolic processes, and is the hub for the interconversion between the three major nutrients. In conclusion, the differentially expressed proteins were mainly concentrated in the energy metabolism and substance synthesis pathways; that is, uranium exposure can lead to abnormal energy metabolism and substance synthesis in HK-2 cells, and the abnormalities in these pathways are closely related to cell apoptosis.

### 3.6. GO and COG Analysis in Proteomics

**Figure 6:**
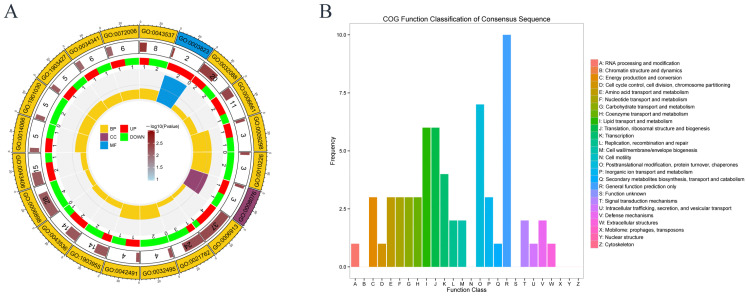
GO and COG analysis. (**A**) Gene Ontology (GO). (**B**) COG annotation.

Gene Ontology (GO) enrichment and Clusters of Orthologous Groups (COG) analysis were subsequently performed. GO is a standard terminology system used to describe the attributes of genes and gene products, which is usually described from three aspects: cellular component (CC), biological process (BP), and molecular function (MF). Figure 6A maps the top 20 GO processes with the smallest *p*-values. From the outside to the inside, the first circle is the GO number, the second circle is how many differentially expressed proteins are related to the GO process, the third circle is the downregulated or upregulated proportion of differentially expressed proteins, and the innermost circle is the GO classification. The differentially expressed proteins were mainly related to biological processes. The most enriched proteins were GO:0006913: nucleoplasmic transport, GO:0006898: receptor-mediated endocytosis, GO:0021762: substantia nigra (brain) development, GO:0032088: negative regulation of NF-KappaB transcription factor activity and GO:0043406: positive regulation of MAPK kinase activity. Uranium is neurotoxic to brain development, and the MAPK pathway is known to be associated with apoptosis induced by uranium [37]. The enrichment proteins in nucleoplasmic transport indicates that uranium may enter the nucleus and thus have an impact on the nucleus. Receptor-mediated endocytosis echoes with the previous KEGG results, which proves that the cytotoxicity caused by uranium may be related to its cellular transport mode.

COG annotation is a method for functional annotation of differential genes. Figure 6B shows that the differential proteins are mainly concentrated in post-translational modification protein folding and chaperone protein, lipid transport and metabolism, translation, ribosome structure and biosynthesis, and transcription process. According to the above analysis, the differential proteins were mainly related to lipid metabolism and protein synthesis, indicating that the damage caused by uranium exposure was related to protein synthesis and lipid metabolism.

### 3.7. Protein–Protein Interaction (PPI) Network of Differentially Expressed Proteins

**Figure 7:**
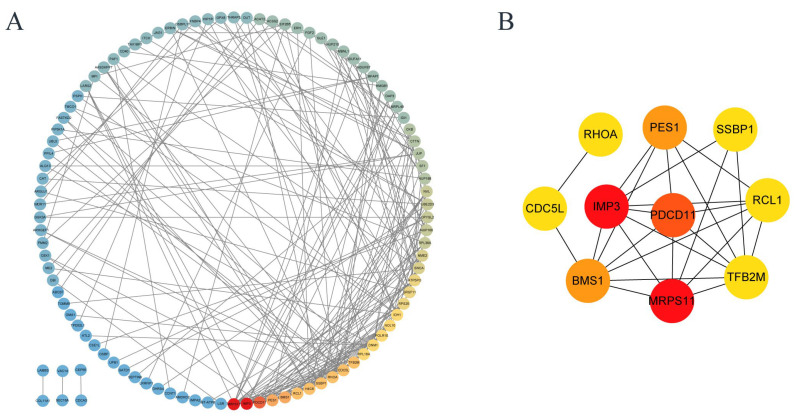
Proteomic interaction network. (**A**) Cyclic graph shows the difference in protein interaction. (**B**) The top ten hub proteins with the closest protein interaction.

Figure 7A shows the differential proteins with interactions to each other, and the color from red to blue shows the interaction density from high to low. The top ten hub differential proteins with the closest protein interaction were selected, and it was found that hub proteins were closely related to ribosomes (Figure 7B and Table 2). The ten proteins with the closest protein–protein interaction relationships show that ribosomal proteins are closely involved in the process of cell damage caused by uranium. In addition, mitochondrial damage and abnormal cell cycle seems to be related to the cytotoxicity of uranium. These results are consistent with the abovementioned conclusions and the reports in the existing literature.

### 3.8. KEGG Enrichment Analysis of Differentially Changed Metabolites in Metabolomics

Metabolomics is the quantitative analysis of all metabolites in an organism and the method used in research to find the relative relationship between metabolites and physiological and pathological changes. The differential metabolites were compared between the control group and the 800 μM group. The screening conditions for DCM were fold change ≥ 2, *p*-value < 0.05, and VIP value ≥ 1.

**Figure 8:**
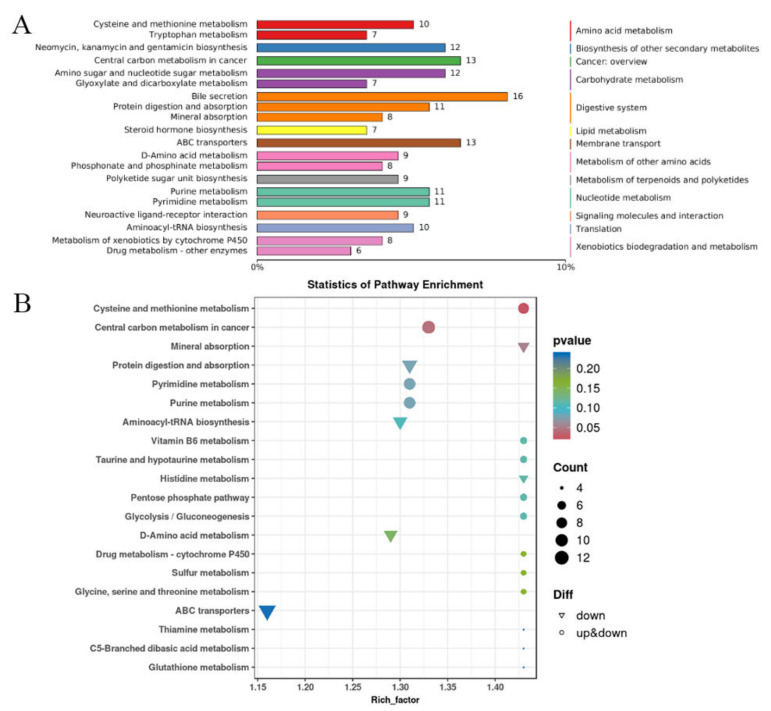
KEGG map of differentially changed metabolites (**A**) KEGG annotation enrichment analysis. The control group and 800 μM group were compared to select differentially changed metabolites. (**B**) KEGG pathway enrichment analysis. Summarizes the significance of top 20 pathways.

Figure 8A indicates that the differential metabolites are mainly concentrated in the bile secretion, central carbon metabolism in cancer, ABC transporters, amino sugar and nucleotide sugar metabolism, purine/pyrimidine metabolism, and aminoacyl-tRNA biosynthesis pathways of cancer. Subsequently, KEGG pathway enrichment analysis was conducted. In Figure 8B, the top 20 significantly enriched pathways are summarized. The size of the points represents the quantity of different metabolites, and the color of the points is related to the statistical credibility of the pathways. The differential metabolites were mainly enriched in ABC transporters, protein digestion and absorption, pyrimidine/purine metabolism, and aminoacyl-tRNA biosynthesis. ABC transporter is a membrane protein that can use ATP energy to transport substances across the membrane, and aminoacyl-tRNA can transfer amino acids to the ribosome to synthesize polypeptide chains. In brief, differential metabolites were related to biosynthesis. This indicates that the impact of uranium on HK-2 cells may mainly be in the aspects of protein and genetic material synthesis and metabolism. In addition, the transport process of uranium may be related to ABC transporter proteins, and the study [38] shows that uranium significantly affects the metabolism of nine amino acids in penicillium, and these amino acids are related to the TCA cycle and ABC transporter proteins.

## 4. Discussion

### 4.1. Uranium Induces Intrinsic Apoptosis in Cells

Intrinsic apoptosis is a programmed cell death pathway triggered by intracellular stress signals. It involves key molecules such as Bcl-2 family proteins, cytochrome C, and the caspase cascade, ultimately leading to cell apoptosis. Uranium exposure can cause DNA damage, mitochondrial dysfunction, and metabolic abnormalities, eventually leading to the occurrence of intrinsic apoptosis.

Uranium induced DNA damage in HK-2 cells. DNA damage is one of the mechanisms of intrinsic apoptosis [39]. Disorders in DNA synthesis and repair are one of the important triggers of apoptosis. For example, mispairing and damage of nucleic acid bases can lead to DNA replication errors, which in turn trigger a DNA damage response that activates the apoptotic program. In the process of apoptosis, DNA fragmentation is one of its characteristic manifestations, which further aggravates the difficulty of DNA synthesis and repair. The results of the comet assay in Figure 3 proved that uranium exposure could cause DNA damage in HK-2 cells. In the KEGG pathway enrichment diagram in Figure 5, pathways related to homologous recombination, mismatch repair, DNA replication, and pyrimidine metabolism are all associated with DNA damage. In the PPI of Figure 7, CDC5L is a protein that is associated with cell cycle regulation and may play a role in DNA replication and repair. SSBP1 is a mitochondrial protein involved in DNA repair and stability maintenance and is important for mitochondrial function. These results suggest that uranium exposure causes DNA damage and therefore may further lead to intrinsic apoptosis.

Mitochondrial dysfunction is one of the important manifestations of energy metabolism imbalance [40]. During apoptosis, cells are in a state of high stress, leading to imbalance of energy metabolism. The lack of energy interferes with the normal physiological activities of cells and prompts them to initiate the apoptotic program. The change in mitochondrial membrane permeability (MOMP) is a key step of mitochondrial induced apoptosis. Bcl-2 family proteins, in which Bax and Bak are activated in response to apoptotic signal stimulation, participate in mitochondrial membrane permeability changes, release cytochrome C, and thus activate the apoptotic program [41]. The decrease in mitochondrial membrane potential leads to potassium efflux, which in turn causes mitochondrial membrane perforation and releases pro-apoptotic factors such as cytochrome C, activates the downstream caspase cascade, and accelerates cell intrinsic apoptosis. Mitochondrial dysfunction can lead to the inhibition of the mitochondrial respiratory chain function, thereby reducing the production of ATP and increasing the levels of ROS. This can further cause increased permeability of the mitochondrial inner membrane, releasing proteins such as cytochrome C from the mitochondria. Cytochrome C binds to the apoptotic protein Apaf-1, forming an apoptosome, which activates caspases and ultimately triggers apoptosis. In addition to this, ROS can affect DNA damage repair, which can also lead to cell apoptosis. ABC transporters (Figure 8B) are responsible for the active transport of a variety of molecules. ABCB7 (Figure 4C) can regulate cell apoptosis by regulating mitochondrial ROS level and NFκB signaling pathway [34]. In addition, the mitochondrial related proteins in Figure 7 are also noteworthy. These results indicate that uranium exposure can cause mitochondrial dysfunction in HK-2 cells, which leads to changes in ATP synthesis and increases ROS production, and further lead to cell intrinsic apoptosis.

Uranium causes metabolic abnormalities that can cause intrinsic apoptosis. During apoptosis, some abnormal synthesis of key proteins may trigger the apoptotic program. In Figure 6, the COG chart indicates that uranium exposure primarily leads to abnormalities in post-translational modification of protein folding and chaperone proteins, lipid transport metabolism, translation, and ribosomal structure and biogenesis. Both Figure 5 and Figure 8 KEGG enrichment diagrams suggest that abnormalities in the synthesis of proteins, nucleotides, and other substances (such as pyruvate metabolism pathway, propionate metabolism pathway, and aminoacyl-tRNA biosynthesis pathway). The PPI (protein–protein interaction) diagram in Figure 7 also shows that the proteins with the closest interactive relationships are related to the processing of ribosomal proteins. Abnormal protein synthesis can lead to apoptosis. Ribosomal dysfunction leads to the release of ribosomal proteins (such as Rpl5, Rpl11, etc.) from the nucleolus into the nucleoplasm [42]. These ribosomal proteins bind to MDM2, inhibiting its E3 ubiquitin ligase activity, thereby preventing the ubiquitination and degradation of p53, which increases the stability and activity of p53. Activated p53 can induce cell cycle arrest or apoptosis. Any impairment in ribosome biogenesis (such as rRNA synthesis, processing, or ribosome assembly) can trigger nucleolar stress [43]. Nucleolar stress activates multiple signaling pathways, leading to cell cycle arrest, senescence, or apoptosis. Nucleolar stress not only activates the p53 pathway but also activates p53-independent DNA damage responses [44]. These stress responses act together to disrupt the cellular environment, ultimately pushing the cells into the apoptosis program. Ribosomal dysfunction results in decreased or abnormal protein synthesis, which in turn affects normal cellular metabolism [44]. In addition, ribosomal dysfunction leads to abnormal protein synthesis and increased oxidative stress in neurons, ultimately resulting in neuronal apoptosis [45]. Therefore, the abnormal metabolism of substances may be one of the mechanisms of intrinsic apoptosis induced by uranium exposure.

### 4.2. Uranium Causes Endoplasmic Reticulum Stress Leading to Cell Apoptosis

Endoplasmic reticulum stress regulates cell survival and death by activating multiple signaling pathways in the unfolded protein response (UPR). Uranium exposure caused changes in UBL5 protein expression, which is associated with ER stress. As shown in Table 1, UBL5 protein expression was significantly decreased following uranium exposure. UBL5 played a protective role in apoptosis induced by endoplasmic reticulum stress, while it was reported that uranium could cause endoplasmic reticulum stress and lead to apoptosis [25]. Therefore, the UBL5 protein is predicted to be associated with uranium toxicological mechanisms. ER-stress-induced UBL5 depletion is mediated by proteasome-dependent but ubiquitin-independent proteolysis. Activation of the protein kinase R-like endoplasmic reticulum kinase (PERK) arm of the UPR is sufficient to induce UBL5 degradation. Silencing of UBL5 leads to the activation of multiple death pathways, which in turn induce apoptosis [46]. When the cell is in a stressed state, UBL5 may determine the fate of the cell through regulating the repair mechanism or the apoptosis initiation mechanism in the cell, that is, whether the cell tries to repair the damage and survive, or initiates the apoptosis program. Therefore, uranium may affect the downstream apoptotic signal transduction by active the ER stress pathway and decrease the UBL5 protein expression and finally leading to cell apoptosis. The ER stress pathway is an important pathway of the intrinsic apoptosis pathway [47], and it is also related to the extrinsic apoptosis. UBL5 is associated with the endogenous pathway of apoptosis which caused by ER stress.

### 4.3. Uranium May Induce Extrinsic Apoptosis in Cells

Extrinsic apoptosis is a programmed cell death pathway triggered by extracellular death signals. This process is usually initiated by the binding of death receptors on the cell surface (such as Fas, TNF receptor) to their ligands (such as FasL, TNF-α). After the ligand binds to the receptor, the receptor undergoes trimerization, recruits adaptor proteins (such as FADD, TRADD), forms the death-inducing signaling complex (DISC), and then activates the initiator caspases (such as caspase-8, caspase-10). These initiator caspases can directly activate downstream effector caspases (such as caspase-3, caspase-7), leading to cell apoptosis. The Fas-mediated apoptotic signaling pathway also involves the activation of a series of downstream molecules, such as JNK, p38-K, and ERK/JNK MAPKs [48]. The activation of these signaling pathways further regulates the apoptotic process of the cell, with JNK (c-Jun N-terminal kinase) being a key branch of the MAPK (mitogen-activated protein kinase) pathway [49]. In Figure 6 of the GO diagram, the MAPK pathway is ranked high in enrichment. Therefore, it is speculated that uranium may induce exogenous apoptosis of HK-2 cells through MAPK pathway. This research provides more evidence indicating that uranium exposure may lead to intrinsic apoptosis rather than extrinsic apoptosis. However, there are still results suggesting that extrinsic apoptosis may also be one of the types of apoptosis caused by uranium exposure.

## 5. Conclusions

Uranyl nitrate at its 24 h IC_50_ (725 µM) commits HK-2 cells predominantly to intrinsic apoptosis, driven by a triad of primary lesions: nuclear DNA damage (increased comet tail length; downregulated CDC5L/SSBP1), mitochondrial dysfunction (loss of ABCB7, decreased ATP, elevated ROS), and endoplasmic reticulum stress (reduced UBL5 expression). Proteome–metabolome integration maps these lesions onto a coherent network in which stalled DNA replication, defective mismatch repair, and pyrimidine imbalance signal through p53-BAX, while UBL5 downregulation amplifies the PERK-CHOP axis; both routes converge on caspase-9 activation. A secondary, weaker extrinsic cue (caspase-8 phosphorylation via MAPK) is detectable but quantitatively minor. Thus, uranium toxicity in proximal-tubular cells is executed chiefly through the intrinsic apoptotic program, with ER stress and marginally extrinsic-driven branches.

## Figures and Tables

**Table 1:** Top 15 differentially expressed proteins (DEPs).

800 μM	Gene/Protein Name	log2FC	Up/Down
Q9BXB4	OSBPL11 (oxysterol-binding protein-related protein 11)	−4.63	down
Q9NWB6	ARGLU1 (arginine- and glutamate-rich protein 1)	−4.48	down
Q15031	LARS2 (leucine–tRNA ligase, mitochondrial)	−4.19	down
Q8NHU6	TDRD7 (Tudor domain-containing protein 7)	−3.64	down
Q8ND56	LSM14A (protein LSM14 homolog A)	−3.42	down
O60563	CCNT1 (cyclin-T1)	−3.29	down
O14960	LECT2 (leukocyte cell-derived chemotaxin-2)	3.19	up
P09038	FGF2 (fibroblast growth factor 2)	3.05	up
Q16890	TPD52L1 (tumor protein D53)	−3	down
Q9NP73	ALG13 (UDP-N-acetylglucosamine transferase subunit ALG13)	2.87	up
Q9ULX9	MAFF (transcription factor MafF)	2.76	up
O00338	SULT1C2 (sulfotransferase 1C2)	−2.54	down
Q9BZL1	UBL5 (ubiquitin-like protein 5)	−2.49	down
Q8NB37	GALD1 (glutamine amidotransferase-like class 1 domain-containing protein 1)	−2.46	down
O75027	ABCB7 (iron–sulfur clusters transporter ABCB7, mitochondrial)	2.46	up

**Table 2:** Top ten hub proteins in proteomic interaction network.

ID	Name	Up/Down
Q9NV31	IMP3 (U3 small nucleolar ribonucleoprotein protein IMP3)	up
P82912	MRPS11 (28S ribosomal protein S11, mitochondrial)	up
Q14690	PDCD11 (protein RRP5 homolog)	up
O00541	PES1 (pescadillo homolog)	up
Q14692	BMS1 (ribosome biogenesis protein BMS1 homolog)	up
Q9Y2P8	RCL1 (RNA 3′-terminal phosphate cyclase-like protein)	up
Q9H5Q4	TFB2M (dimethyladenosine transferase 2, mitochondrial)	up
P61586	RHOA (transforming protein RhoA)	down
Q99459	CDC5L (cell division cycle 5-like protein)	down
Q04837	SSBP1 (single-stranded DNA-binding protein, mitochondrial)	up

## Data Availability

Data will be made available on request.
